# From Psychogenic Cough to Somatic Cough Syndrome

**DOI:** 10.1111/crj.70152

**Published:** 2026-01-05

**Authors:** Haiyang Wang, Tongyangzi Zhang, Li Yu, Xianghuai Xu

**Affiliations:** ^1^ Department of Pulmonary and Critical Care Medicine, Tongji Hospital, School of Medicine Tongji University Shanghai China; ^2^ Department of Allergy, Tongji Hospital, School of Medicine Tongji University Shanghai China

**Keywords:** chronic cough, psychogenic cough, psychological disorders, somatic cough syndrome, somatic symptom disorders

## Abstract

Patients with various etiologies of chronic cough may have psychological disorders, and those with psychological disorders may also exhibit physical symptoms such as coughing. The terminology “psychogenic cough” and “somatic cough syndrome” have been used to describe chronic cough patients with underlying psychological issues, but both terms lack clear definitions and diagnostic criteria. This article provides a review of the definition changes, pathogenesis, and diagnostic and therapeutic points related to chronic cough with comorbid psychological disorders based on relevant studies of psychogenic cough and somatic cough syndrome.

## Graphical abstract

This review explores the evolution from “Psychogenic cough” to “Somatic cough syndrome” in chronic cough patients with underlying psychological issues. It highlights the complex, bidirectional relationship between chronic cough and psychological disorders, and discusses updated diagnostic criteria and management strategies, advocating for a multidisciplinary approach to improve patient care.

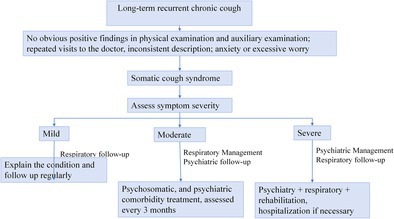

## 1 Introduction

Chronic cough is one of the most common reasons for seeking medical care in respiratory medicine. It typically refers to a cough lasting more than 8 weeks, in which coughing is the primary or sole symptom, and no significant abnormalities are detected on chest radiographs [1]. The causes of chronic cough are complex and may include various respiratory and nonrespiratory conditions, such as upper airway cough syndrome, cough‐variant asthma, and gastroesophageal reflux disease. Persistent coughing not only affects quality of life but can also lead to sleep disturbances, limitations in social activities, and increased psychological stress [2].

In recent years, the role of psychological factors in the development and persistence of chronic cough has drawn increasing attention. Studies have shown that one‐third of patients with chronic cough present with psychiatric comorbidities such as anxiety and depression [3]. Psychogenic cough (PC) has traditionally been utilized to describe patients who suffer from both psychological disorders and persistent coughing. Initially, it was assumed that underlying psychological issues were the primary cause of the persistent cough, with a PC diagnosis only made after thorough examinations ruled out physical illnesses. However, the newly proposed term “somatic cough syndrome (SCS)” suggests that chronic cough may not necessarily have a direct cause‐and‐effect relationship or temporal sequence [4]. The introduction of the SCS emphasizes the importance of how patients perceive and experience cough symptoms, rather than emphasizing their correlation with psychological issues. This paper reviews relevant research on PC and SCS, with a focus on the relationship between chronic cough and psychological disorders.

## 2 Literature Search

The search was conducted until May 1, 2025. Two independent authors searched the PubMed, Scopus, and Web of Science databases for studies that provided information about the relationship between chronic cough and psychological problems. To cover all relevant areas, a complex search strategy was developed using not only the term “chronic cough” but also terms thought to be associated with cough. These were “chronic obstructive pulmonary disease,” “asthma,” “pneumonia,” “respiratory infections,” “carcinoma,” “bronchogenic,” “gastro‐oesophageal reflux,” and “angiotensin converting enzyme inhibitors.” To identify studies reporting on cough with psychological problems, we added the terms “psychological problems,” “psychological disorders,” “psychogenic cough,” “somatic cough syndrome,” and “somatic symptom disorder.” Wherever possible, Medical Subject Headings terms were used. The starting date for the search set was 1963, as this is the first year from which a sufficient number of studies were available.

## 3 From Psychogenic Cough to Somatic Cough Syndrome

PC has a long historical background, with the earliest case descriptions dating back to 17th‐century medical literature [5, 6]. Its main characteristics include the disappearance of cough during sleep, daytime prominence, and association with emotional changes [7, 8, 9]. By the mid‐20th century, PC was gradually applied to the diagnosis of chronic cough in children and adolescents, emphasizing the dominant role of psychological factors in symptom formation. In addition to PC, other clinical terms commonly used during this period included functional cough, psychological cough, hysterical cough, tic cough [10], vocal tic, Tourette syndrome, involuntary cough syndrome, crowing cough, and barking cough. Although these terms varied in name and had different definitions and diagnostic criteria, they all referred to chronic cough related to psychological and emotional factors. According to statistics, as of 2014, 90% of related studies adopted the term “psychogenic cough” [11, 12].

In the past, the International Classification of Diseases, 10th Revision (ICD‐10) and earlier diagnostic systems included PC in the category of somatoform disorders. However, this diagnosis has many shortcomings in clinical practice, such as overly vague definitions and strict criteria, and the difficulty of accepting the “mind‐body dualism” that separates organic diseases from psychological states [13, 14]. Therefore, the latest ICD‐11 and the Diagnostic and Statistical Manual of Mental Disorders, 5th edition (DSM‐5) propose Somatic Symptom Disorder (SSD), which refers to the existence of one or more physical symptoms that cause significant distress or impairment in daily life [15]. At the same time, the patient has excessive or disproportionate abnormal thoughts, feelings, cognition, or behaviors related to physical symptoms, or excessive emotional experiences related to health. The diagnosis no longer emphasizes whether the patient's physical symptoms can be explained by organic diseases but focuses on the patient's excessive attention to their own physical symptom [16]. To be consistent with the DSM‐5, the ACCP recommended in 2014 that SCS be used in new cough guidelines to replace PC [4].

“Habit Cough (HC)” is commonly used to describe a characteristic cough that persists after infection without an organic basis. First proposed by Berman in 1966, this term remains in use for diagnosing children with chronic cough after excluding organic causes [9, 17, 18]. HC emphasizes the role of behavioral factors in cough persistence without necessarily linking it directly to psychological disorders. The core distinctions among HC, PC, and SCS lie primarily in their etiological attribution, diagnostic criteria, and theoretical foundations. These differential points are systematically contrasted in Table 1. While PC emphasizes psychological conflicts as the dominant factor in cough manifestation, HC focuses on the behavioral reinforcement of cough patterns and is primarily applied to pediatric cases. In contrast, SCS approaches persistent cough from a psychosomatic perspective, explaining symptom maintenance through mind–body interactions. Consequently, in the management of chronic cough in adults, SCS demonstrates better alignment with contemporary psychosomatic medicine principles and offers greater clinical applicability.

**TABLE 1:** Comparison of functional or psychological terms related to chronic cough.

	Typical age group	Key characteristics	Relationship with psychological factors
HC	Children, adolescents	Dry, barking cough; disappears during sleep; often follows an upper respiratory tract infection	A clear causal relationship is often not emphasized; considered an involuntary “habit”
PC	Predominantly children (6–14 years), rare in adults	Daytime cough, absent during sleep; often accompanied by anxiety	Traditionally considered that psychological issues are the primary cause of cough (an exclusion diagnosis)
SCS	All age groups, more prominent in adults	Cough as the primary somatic symptom; accompanied by excessive concerns/worries and behaviors related to the symptom/health	Based on the DSM‐5/ICD‐11, emphasizing cognitive, emotional, and behavioral abnormalities related to symptoms

Abbreviations: HC, habit cough; PC, psychogenic cough; SCS, somatic cough syndrome.

The definition of chronic cough with comorbid psychological disorders varies among different countries' cough guidelines. The latest Chinese cough guideline [1], French adult chronic cough guideline [19], and European Respiratory Society guideline [20] have all introduced the concept of SCS. The latest Australian guidelines have adopted the concepts of both HC and SCS [21]. Whereas the cough guidelines of Japan [22] and South Korea [23] have not yet introduced this concept and still refer to it as HC or PC.

## 4 Etiology and Pathogenesis

The literature suggests that the pathogenesis of PC involves multiple factors, of which psychological factors are only a part [15]. Abnormalities in central nervous system regulation and emotional disorders are also important influencing factors. Patients are often unable to express their inner discomfort properly and instead translate it into physical reactions, such as cough [24]. Children are particularly vulnerable to external pressures such as family conflicts and learning difficulties, which can lead to PC.

The pathogenesis of SCS is mainly based on research into SSD and PC and is explained by the biopsychosocial model of disease, which suggests that several factors contribute to its development.

Firstly, biological factors play a role in SCS. It is associated with an imbalance in the brain's cortical control of cough, characterized by an over‐sensitivity of the central nervous system to cough stimuli [25]. This hypersensitivity is a major cause of chronic cough, especially treatment‐resistant chronic cough and cough hypersensitivity syndrome [26]. When the central nervous system is oversensitive to cough stimuli, even mild or normal peripheral nerve stimulation can trigger or exacerbate cough reflexes [27]. Functional magnetic resonance imaging (fMRI) has shown that the brain regions responsible for controlling and regulating respiratory movements change during active suppression of cough [28]. This region overlaps with the area associated with psychological factors, possibly located in the left frontal lobe [29]. However, direct neuroimaging studies specifically in patients diagnosed with SCS are still lacking and represent a critical area for future research.

Secondly, psychological factors may also contribute to SCS. Patients with certain personality traits, such as adverse personality traits, avoidance or anxiety attachment patterns, and abnormal emotional cognitive regulation (such as affective disorders), are more prone to various physical symptoms [30, 31]. These physical symptoms may manifest as specific sensations (such as pain or coughing) or systemic symptoms (such as fatigue or weakness), which are not related to known medical causes or are much more severe than expected. In addition, some patients may have negative beliefs and cognition, such as excessive sensitivity or fear of drugs or the environment, which may cause them to pay excessive attention to or exaggerate their respiratory sensations, thereby inducing or maintaining chronic coughing.

Finally, social factors may also contribute to the development of SCS. Life event stress and extensive psychosocial stressors, such as family changes, acute physical illnesses, and stressful work environments, are important factors in inducing physical discomfort [32]. These factors may cause excessive nervous system tension, interfere with emotional cognitive regulation, and make it difficult for patients to express and process their emotional issues. When emotional stress reaches a certain level, patients may unconsciously transform it into physical reactions, resulting in various unexplained physical symptoms.

## 5 Clinical Characteristics

PC is common in children aged 6 to 14 years [12] and is characterized by coughing as the main symptom. It typically presents as daytime cough that resolves during sleep and is often associated with anxiety symptoms [33]. Although less common in adults, PC also has the characteristic of daytime coughing disappearing during sleep [34]. The main trigger factors are upper respiratory tract infections, and children often develop a habit of coughing after illnesses such as colds, eventually becoming dependent on the coughing symptom, even if the original disease has been cured [11]. It has been reported that the most commonly associated mental disorders with PC include conversion disorder (22%), anxiety and depression mixed disorder (12%), and hereditary anxiety disorder (10%) [35]. It is worth noting that daytime coughing, as well as barking or honking coughs, is not specific clinical feature of this disease, and other diseases, such as coughs caused by atypical pathogens, can also have these characteristics. Therefore, the coughing characteristics are only indicative of PC and do not have a diagnostic role [4].

The clinical manifestations of SCS are similar to those of PC, but the patient's excessive focus on their own symptoms or health is more apparent. This includes obsessing about symptoms or health‐related thoughts, worrying about being ill, and repeatedly seeking diagnosis through compulsive behavior, which not only interferes with daily life but also often leads to tense doctor‐patient relationships during treatment. The most common accompanying symptoms reported are an itchy throat (60.9%), followed by throat‐clearing (47.8%), postnasal drip (47.8%), foreign body sensation in the throat (39.1%), chest tightness (39.1%), and shortness of breath (30.4%) [36].

Laboratory testing for PC and SCS lacks specific biological indicators, and physical and auxiliary examinations, including chest imaging, pulmonary function tests, allergen tests, 24‐h multichannel oesophageal impedance monitoring combined with pH monitoring, and bronchoscopy, have not shown clear positive findings [1, 37]. Recording the characteristics of coughing with instruments is a potentially effective detection method, but further research is needed to validate it. Some scholars have used a simple cough counting device to record the coughing situation of suspected PC patients throughout the day, proving that the coughing significantly decreases when the patient is asleep and more awake, and cough waveform analysis indicates that most coughs only have the first sound [38]. Imai's study [39] also confirmed this, showing that the cough frequency is very high while awake (80 times in 30 min), but is significantly reduced during sleep. Furthermore, the nature of the cough during sleep differs from that in the waking state.

## 6 Diagnostic Criteria and Assessment Methods

### 6.1 Diagnostic Criteria

The diagnosis of PC is an exclusionary diagnosis with no specific diagnostic criteria. Only after exclusion of common and rare causes of chronic cough can this diagnosis be considered [1]. Based on a synthesis of previous literature perspectives [4, 12, 37, 40], the diagnosis of PC is highly dependent on the clinical presentation. If a patient's cough symptoms cannot be explained by other medical conditions (such as asthma, sinusitis, and gastroesophageal reflux disease) or medications (such as ACE inhibitors), are related to stress and anxiety, or respond to psychiatric treatment, a diagnosis of PC may be considered.

SCS can only be diagnosed after excluding common causes of cough and upon meeting the diagnostic criteria for SSD through a joint evaluation by the psychosomatic department [4]. According to DSM‐5 [15], the diagnostic criteria for SSD (see Table S1) include three aspects: Criterion A: one or more distressing somatic symptoms such as cough; Criterion B: excessive thoughts, feelings, and behaviors about the symptoms or health problems, such as worrying about having a serious respiratory disease; and Criterion C: the time spent being bothered by one or more symptoms is more than 6 months. In addition, SSD can be classified into different levels of severity depending on the severity of the patient's symptoms, psychological characteristics, and functional impairment. Diagnosis of SSD usually requires a consultation with a psychosomatic physician and must be in accordance with ICD‐11. It often requires a structured clinical interview for DSM‐5 (SCID‐5) [41] to systematically gather information about the patient's psychological and physical aspects, to assess the patient's perception of the symptoms, emotional and behavioral impact, and the communication style between the patient and the clinician.

Unlike the purely exclusion‐based diagnosis of PC, SCS focuses on the maladaptive thoughts, feelings, and behaviors surrounding the cough symptom. However, the current diagnostic paradigm for SCS still necessitates the exclusion of alternative organic etiologies before it can be formally applied. While valuable, the current exclusive approach could be enhanced to better reflect the widespread nature of cough‐psychological comorbidity. Many patients with clear organic triggers (e.g., asthma and reflux) concurrently develop significant psychological distress about their cough. The present model may not fully account for this overlap. Evolving the SCS construct by relaxing the exclusion prerequisite could thus offer a more powerful and broadly applicable tool for identifying and describing this comorbidity in diverse clinical presentations.

### 6.2 Assessment

The use of measurement tools to diagnose psychiatric disorders is a convenient and effective way to quickly screen and assess a patient's physical and psychological symptoms and quality of life, and to monitor treatment outcomes. Until now, no specific assessment method for PC has been available [37]. Instead, scales to assess SCS, based on the diagnostic criteria for SSD in the DSM‐5, are divided into two main categories, assessing patients' somatic symptom load and psychological symptom burden, respectively, and have been widely used.

Scales for measuring the somatic symptom burden of physical symptoms related to Criterion A include (1) the PHQ‐15, which is suitable for screening patients with mild to moderate SSD and can also be used to assess the impact of physical symptoms on quality of life and function [42]; (2) the Somatic Symptom Scale‐8 (SSS‐8) (see Table S2), a brief self‐report scale that assesses the perceived burden of common physical symptoms, including headache, chest tightness, abdominal discomfort, constipation, joint pain, and other bodily complaints [43]. It is characterized by simplicity, ease of use, high reliability, and validity, and can be used in different cultural backgrounds and clinical settings.

The scales used to measure the burden of psychological symptoms related to Criterion B include the SSD‐12 (see Table S3), a brief self‐report questionnaire designed to assess a patient's thoughts, feelings, and behaviors related to their symptoms. It consists of 12 items, each of which is scored on a scale of 0 to 4 (0 = *never*, 1 = *rarely*, 2 = *sometimes*, 3 = *often*, 4 = *very often*). The scores are added to give a simple total score (which can range from 0 to 48). Its advantages are ease of completion, a simple scoring algorithm (addition of responses), and ease of interpretation [44].

## 7 Treatment

### 7.1 Nonpharmacological Treatment

The main treatment for PC is psychological intervention, including suggestion, self‐hypnosis, and cognitive‐behavioral therapy (CBT), and drug treatment is generally not recommended unless there are obvious anxiety or depressive symptoms, in which case antianxiety or antidepressant medications may be used appropriately [40].

Suggestion therapy is the preferred method for treating PC in children and can quickly relieve or eliminate coughing in most cases [45]. Studies by Weinberger et al. [17] and Haydour et al. [11] have confirmed the effectiveness of suggestion intervention in resolving coughing problems in PC patients (*n* = 52). This method includes education, calming, practice, guidance, and psychological education [46, 47]. Interestingly, Weinberger also found that watching videos of other patients who had been successfully treated with suggestion therapy could also reduce one's cough frequency [48].

CBT, widely used for a variety of psychological disorders, has also been applied to PC, particularly for patients with associated anxiety traits or maladaptive cough‐related beliefs [49]. Although large‐scale controlled trials are limited, available case reports and small clinical series indicate that CBT can reduce cough frequency and improve coping strategies by targeting dysfunctional cognitions and reinforcing behavioral control techniques [50].

Self‐hypnosis is an important supplement in clinical practice. Anbar has conducted a number of studies over the years and found that hypnosis can effectively help children with respiratory diseases control discomfort and reduce the number of analgesics they need [51, 52, 53, 54]. The retrospective study by Haydour et al. [11] also supports this view and found that 78% of patients who received hypnosis treatment experienced cough relief.

Treatment for SCS lacks evidence from clinical trials and is mostly psychotherapeutic [55, 56] and focuses on changing the way patients perceive and control their symptoms through therapy, including (1) CBT: This therapy aims to change the way patients interact with their symptoms by altering their thoughts and behaviors, and providing them with positive coping strategies. One study reported significant symptom improvement in a 10‐year‐old patient with SCS after receiving CBT [57]. (2) Mindfulness‐based therapy (MBT): This therapy helps patients develop a new attitude towards their symptoms, promoting nonjudgmental acceptance of physiological or psychological pain. (3) Psychodynamic therapy: This therapy aims to reveal unconscious content, identify, and address the causes and sustaining factors of symptoms. (4) Family therapy: This therapy uses the family as the intervention unit to eliminate abnormal behaviors and physiological functions, promoting individual and family system functioning. (5) Other methods, such as hypnosis, behavioral, humanistic, and integrative approaches, require further evidence to support their effectiveness. Some patients may be unwilling to undergo psychotherapy and may opt for enhanced conventional treatment [55, 58].

### 7.2 Pharmacological Treatment

There are no drugs available for the pathogenesis of PC, and the appropriate drugs are selected clinically according to their degree of cough and psychiatric symptoms [1].

For SCS, there is currently a lack of high‐quality evidence to support any particular treatment method, so more clinical research is needed to verify the effectiveness and safety of these methods [4]. Clinically, appropriate medications are selected based on the patient's physical and psychological symptoms, such as medications that relieve physical symptoms and Traditional Chinese Medicine (TCM). If the patient has obvious anxiety and depression symptoms, psychiatric drugs such as antianxiety, antidepressant, and antipsychotic drugs can be used for treatment [59]. TCM can also be used to treat patients' physical discomfort or functional disorders. Common external treatment methods include acupuncture, massage, cupping, and scraping [60]. Internal herbal formulations such as maekmoondong‐tang are also commonly used [61]. Other auxiliary methods may be used for the patient's lifestyle or social support, such as exercise, relaxation training, sleep hygiene advice, and dietary supplements.

## 8 Relationship Between Chronic Cough and Psychological Disorder

Chronic cough is a serious respiratory symptom that has a significant impact on a patient's quality of life. There are many possible causes, including infection, allergies, airway hyperresponsiveness, and gastroesophageal reflux. However, some patients, even after thorough examination and treatment, still experience no significant improvement and are diagnosed with unexplained chronic cough (UCC). UCC may be closely related to psychological factors [25], such as various internal or external factors that affect an individual's emotions, cognition, and behavior, including stress, emotions, personality, and beliefs.

**FIGURE 1:**
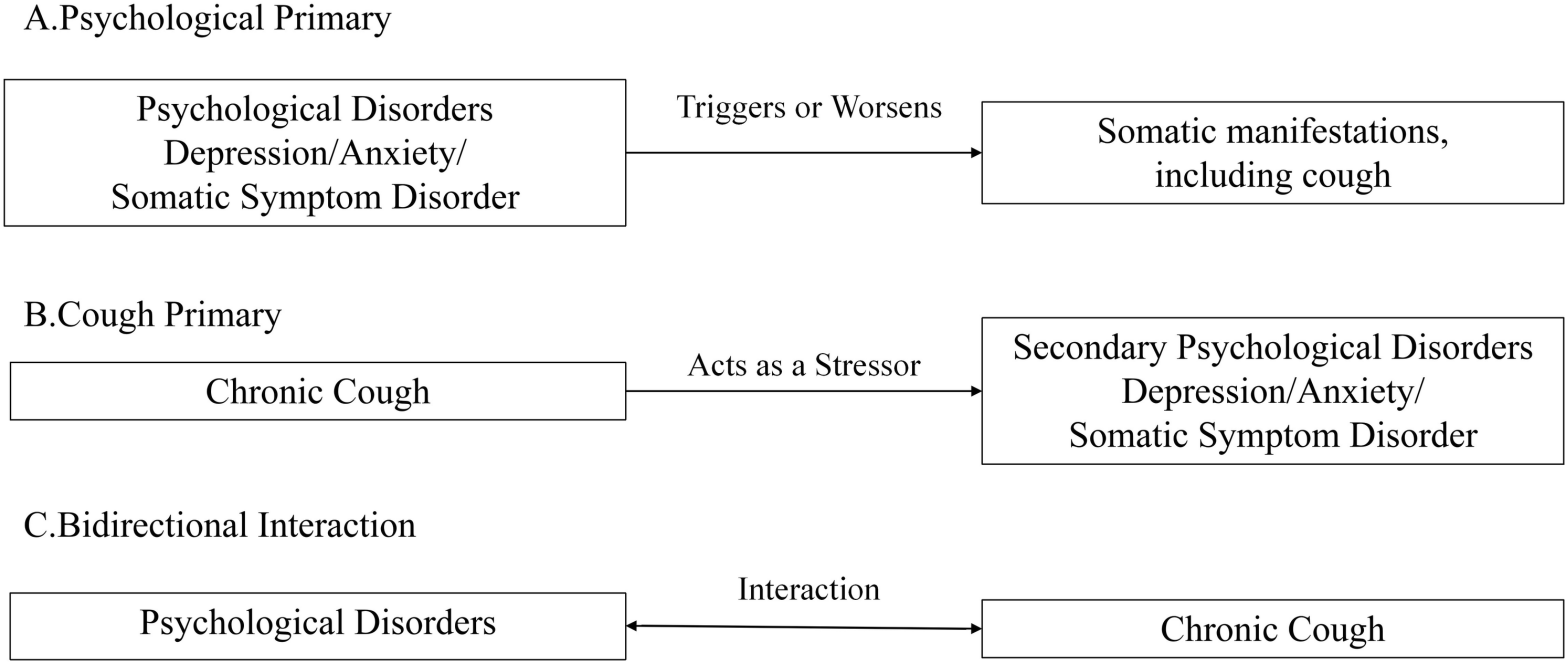
A hypothesis about the relationship between chronic cough and psychological disorder.

Regarding the relationship between chronic cough and psychological problems, the ACCP Expert Panel proposed three possible relationships [37]: (A) Coughing is the result of psychological problems, and cough symptoms may improve after successful treatment of these issues; (B) coughing is the cause of psychological problems and appears alongside chronic cough, but improvement may be achieved after successfully treating the cough; and (C) coughing and psychological problems coexist, and there is a bidirectional causal relationship between them. Figure 1 illustrates the relationship that requires further research.

Coughing may be the result of psychological problems. Psychological factors can affect the body through the neuroendocrine‐immune system, causing or aggravating certain physical conditions, such as hypertension, coronary heart disease, and gastric ulcers. Similarly, psychological factors can also affect the respiratory system, causing or exacerbating airway inflammation, airway hyperresponsiveness, and airway nerve sensitivity, leading to or exacerbating chronic cough. A multicenter study of 4762 adults found that 9.3% of people had respiratory‐related physical symptoms [62], and the incidence of such symptoms is higher in patients with psychological disorders. A multicenter study in China suggested that the proportion of respiratory‐related physical symptoms in patients with severe depression was about 29.6% [63]. Our previous research found that some patients with psychological disorders develop cough symptoms after being diagnosed with psychological symptoms or disorders [64]. Compared with other patients with chronic cough, the psychological assessment results of these patients suggest that their psychological problems are more severe and their cough‐related quality of life is lower.

Chronic cough can be the cause of psychological issues. Chronic cough itself is a distressing and bothersome symptom that can affect the patient's sleep, work, social life, and overall quality of life, reducing their self‐efficacy. Long‐term discomfort and ineffective treatment can cause patients to develop negative emotions such as helplessness, frustration, and anxiety, which may further develop into depression or anxiety disorders. Previous studies have shown that anxiety and depression are common psychological issues among chronic cough patients and are positively correlated with the severity and duration of coughing [36]. This is consistent with our previous research [64], which found that some chronic cough patients develop anxiety or depression after the onset of coughing, which may be related to the course and cause of coughing. In addition, some physical illnesses may also cause or exacerbate psychological problems by affecting respiratory function. For example, gastroesophageal reflux disease and chronic obstructive pulmonary disease are both conditions that can cause chronic cough symptoms, and studies have found that patients with these diseases have significantly higher levels of anxiety and depression [65, 66].

There may be a bidirectional causal relationship between chronic cough and psychological issues, rather than a unidirectional one. This perspective suggests that chronic cough is not only a symptom of respiratory disease but also an expression of psychological problems. Chronic cough can lead to psychological barriers such as anxiety, depression, and low self‐esteem, and these psychological barriers may exacerbate or perpetuate chronic cough. SCS is an approach that considers and treats chronic cough and psychological issues as a whole, without denying the organic basis of chronic cough or ignoring the impact of psychological factors on it. In contrast, PC is an exclusive diagnosis that can only be considered after ruling out all possible organic causes. The treatment of PC mainly relies on suggestion therapy, psychological intervention, and antianxiety medication, while the treatment of SCS may require the comprehensive use of drugs, physical, behavioral, and psychological methods to control inflammation, relieve irritation, improve respiratory function, regulate emotions, enhance confidence, and achieve multiple effects. Therefore, SCS may better align with the complexity and multifactorial nature of chronic cough, potentially helping patients improve their quality of life and increase the cure rate.

It must be recognized that psychological disorders such as depression and anxiety may be causes of cough but are more likely to be direct consequences of unresolved chronic cough. Therefore, it should not be assumed that all cases of cough accompanied by psychological symptoms are psychogenic. Adhering to the DSM‐5 criteria and accurately using terms such as SCS can help avoid misdiagnosing coughs with organic etiology that are accompanied by psychological distress, thereby ensuring the correct treatment direction [67].

## 9 Conclusion

**FIGURE 2:**
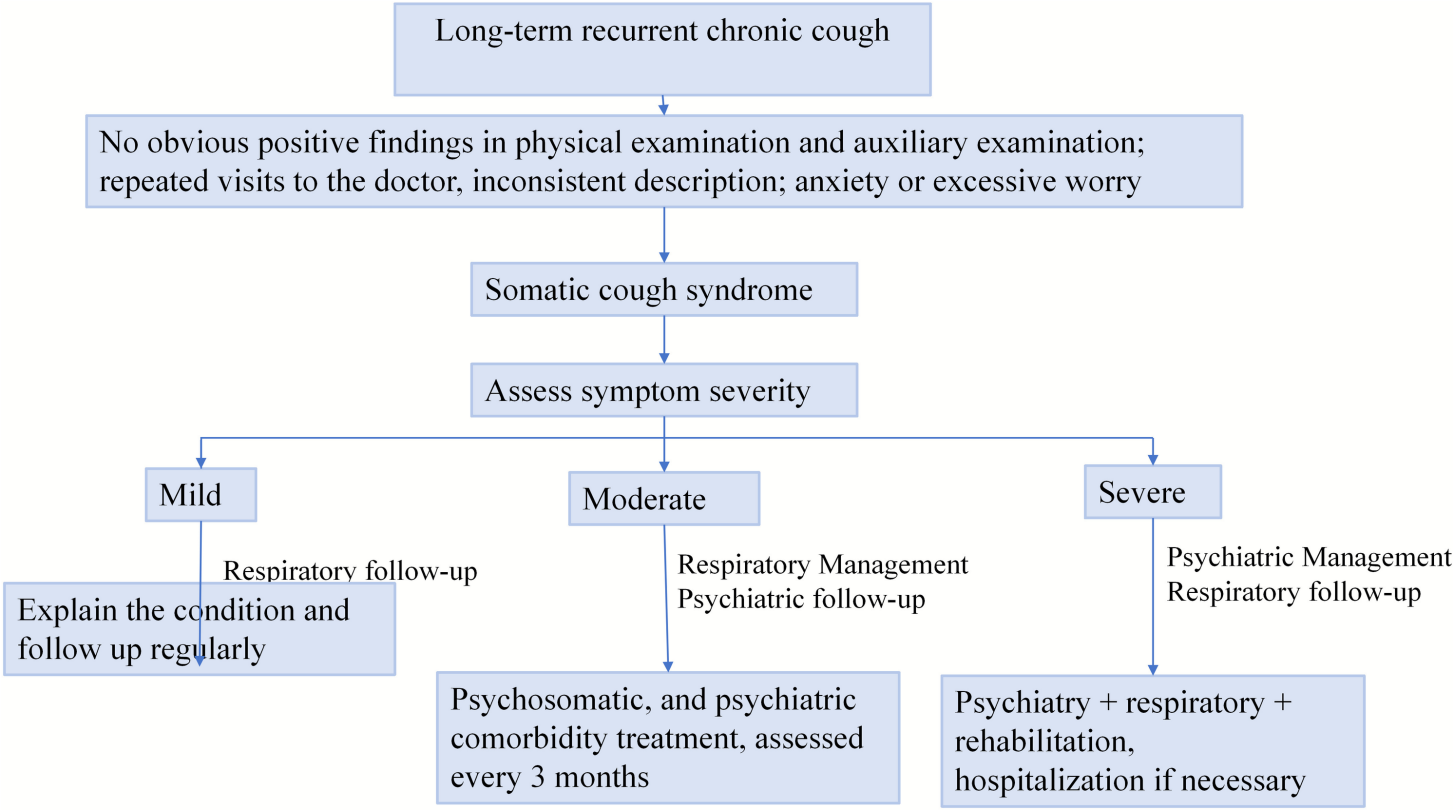
The treatment process of somatic cough syndrome.

There is a complex and intimate relationship between chronic cough and psychological factors, which may be either cause, effect, or reciprocal. Although this relationship is difficult to distinguish clearly, relevant research remains essential. SCS provides a valuable framework for this, characterizing a condition primarily manifested as chronic cough, where the distress and impairment stem predominantly from the patient's excessive thoughts, feelings, and behaviors related to the symptom. In recent years, the diagnostic conceptualization has beneficially expanded from the purely exclusion‐based PC to the more positive and comprehensive SCS construct. This expansion facilitates more accurate patient identification, enhances clinical awareness, and promotes essential multidisciplinary management, as summarized in Figure 2. It should be acknowledged, however, that the current reliance of SCS diagnosis on excluding organic pathologies may not adequately address the psychological distress in patients who have comorbid organic conditions. Future studies should refine the diagnostic criteria for SCS to move beyond reliance on exclusion‐based methods, better capturing its psychosomatic nature and enhancing its clinical utility.

## Author Contributions

H.W. and T.Z. contributed equally to this paper and are joint first authors. H.W. and T.Z. contributed to study concept and design, analysis and interpretation of data, and drafting of the manuscript. X.X. and L.Y. contributed to data acquisition and critical revision of the manuscript.

## Funding

This study was supported by the National Natural Science Foundation of China (No. 82270114 and 82570146), the Project of Science and Technology Commission of Shanghai Municipality (No. 22Y11901300, 21Y11901400, and 20ZR1451500), the Program of Shanghai Academic Research Leader (No. 22XD1422700), and the Fund of Shanghai Youth Talent Support Program.

## Ethics Statement

The authors have nothing to report.

## Consent

The authors have nothing to report.

## Conflicts of Interest

The authors declare no conflicts of interest.

## Supporting information


**Table S1:** Diagnostic criteria for somatic symptom disorders.
**Table S2:** Somatic symptom scale SSS‐8.
**Table S3:** Somatic symptom disorder B standard scale.

## Data Availability

No data are available.
