# Supplementary material for: From Psychogenic Cough to Somatic Cough Syndrome
Source: Clin Respir J. 2026 Jan 5;20(1):e70152. doi: 10.1111/crj.70152 (PMC12771670; doi:10.1111/crj.70152)
Supplement: Supplementary file 1 — Table S1: Diagnostic criteria for somatic symptom disorders. Table S2: Somatic symptom scale SSS‐8. Table S3: Somatic symptom disorder B standard scale. [file CRJ-20-e70152-s001.pdf]

Appendix:

Supplementary Table S1: Diagnostic criteria for somatic symptom disorders

Diagnostic criteria
A. One or more physical symptoms that are distressing or cause disruption in daily life
B. Excessive thoughts, feelings or behaviors related to the physical symptoms or health concerns with at least one of the following:
1. Ongoing thoughts that are out of proportion with the seriousness of symptoms
2. Ongoing high level of anxiety about health or symptoms
3. Excessive time and energy spent on the symptoms or health concerns
C. Although any one somatic symptom may not be continuously present, the state of being symptomatic is persistent (typically more than 6 months).
Severity
Mild: Only one of the symptoms specified in Criterion B is fulfilled.
Moderate: Two or more of the symptoms specified in Criterion B are fulfilled.
Severe: Two or more of the symptoms specified in Criterion B are fulfilled, plus there are multiple somatic complaints (or one very severe somatic symptom).

Supplementary Table S2: Somatic symptom scale SSS-8

During the past 7 days, how much have you been bothered by any of the following problems?					
	Not at all	A little bit	Somewhat	Quite a bit	Very much
Stomach or bowel problems	4	3	2	2	0
Back pain	4	3	2	2	0
Pain in your arms, legs, or joints	4	3	2	2	0
Headaches	4	3	2	2	0
Chest pain or shortness of breath	4	3	2	2	0
Dizziness	4	3	2	2	0
Feeling tired or having Low energy	4	3	2	2	0
Trouble sleeping	4	3	2	2	0
**Severity categories:**					
0-3（No to minimal）					
4-7（Low）					
8-11（Medium）					
12-15（High)					
16-32（Very high）					

Supplementary Table S3: Somatic symptom disorder B standard scale

Index	Symptom
1	I think that my physical discomfort is a harbinger of a serious illness
2	I'm worried about my health.
3	My concerns about my health are getting in the way of my daily life

4	I'm pretty sure my somatic discomfort is serious
5	My physical discomfort scares me
6	For most of the day I am plagued by physical discomfort
7	Others told me. My somatic discomfort well not serious
8	I worry that my somatic discomfort is endless
9	I am exhausted by my concerns about my health
10	I think my doctors don't pay enough attention to my somatic discomfort
11	It is difficult for me to focus on other things because of my discomfort
12	I am concerned that the effects of my discomfort will continue to affect me in the future
